# 2-Aminoterephthalic acid dimethyl ester

**DOI:** 10.1107/S1600536809036095

**Published:** 2009-09-16

**Authors:** Jürgen Brüning, Jan W. Bats, Martin U. Schmidt

**Affiliations:** a Institute of Inorganic and Analytical Chemistry, University of Frankfurt, Max-von-Laue-Strasse 7, D-60438 Frankfurt am Main, Germany; b Institute of Organic Chemistry and Chemical Biology, University of Frankfurt, Max-von-Laue-Strasse 7, D-60438 Frankfurt am Main, Germany

## Abstract

Single crystals of the title compound, C_10_H_11_NO_4_, an inter­mediate in the industrial synthesis of yellow azo pigments, were obtained from the industrial production. The mol­ecules crystallize as centrosymmetic dimers connected by two symmetry-related N—H⋯O=C hydrogen bonds. Each mol­ecule also contains an intra­molecular N—H⋯O=C hydrogen bond. The dimers form stacks along the *a*-axis direction. Neighbouring stacks are arranged into a herringbone structure.

## Related literature

For studies on amino­terephthalic acid esters, see: Wegscheider *et al.* (1912 ▶); Clark *et al.* (1995 ▶); O’Connor *et al.* (1999 ▶); Lavalette *et al.* (2002 ▶); Jones *et al.* (2008 ▶). For syntheses wherein the title compound is used, see: Cordier & Coulet (1994 ▶); Metz & Weber (1999 ▶); Stengel-Rutkowski & Metz (2000 ▶); Jung *et al.* (2001 ▶); Herbst & Hunger (2004 ▶); Schweikart *et al.* (2007 ▶). For the crystal structure of the final product, Pigment Yellow 213, see: Schmidt *et al.* (2009 ▶).
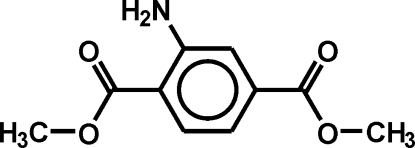


## Experimental

### 

#### Crystal data

C_10_H_11_NO_4_
*M* *_r_* = 209.20
Monoclinic, 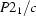

*a* = 4.7721 (12) Å
*b* = 16.928 (5) Å
*c* = 11.841 (5) Å
β = 93.88 (5)°
*V* = 954.4 (6) Å^3^
*Z* = 4
Mo *K*α radiation
μ = 0.11 mm^−1^
*T* = 166 K
0.75 × 0.32 × 0.04 mm

#### Data collection

Siemens SMART 1K CCD diffractometer
Absorption correction: multi-scan (*SADABS*; Bruker, 2000 ▶) *T*_min_ = 0.760, *T*_max_ = 0.995
11829 measured reflections
1687 independent reflections
884 reflections with *I* > 2σ(*I*)
*R*_int_ = 0.140

#### Refinement

*R*[*F* ^2^ > 2σ(*F* ^2^)] = 0.054
*wR*(*F* ^2^) = 0.134
*S* = 0.96
1687 reflections
146 parameters
H atoms treated by a mixture of independent and constrained refinement
Δρ_max_ = 0.27 e Å^−3^
Δρ_min_ = −0.24 e Å^−3^

### 

Data collection: *SMART* (Siemens, 1995 ▶); cell refinement: *SAINT* (Siemens, 1995 ▶); data reduction: *SAINT*; program(s) used to solve structure: *SHELXS97* (Sheldrick, 2008 ▶); program(s) used to refine structure: *SHELXL97* (Sheldrick, 2008 ▶); molecular graphics: *Mercury* (Macrae *et al.*, 2008 ▶); software used to prepare material for publication: *publCIF* (Westrip, 2009 ▶).

## Supplementary Material

Crystal structure: contains datablocks I, global. DOI: 10.1107/S1600536809036095/fk2004sup1.cif

Structure factors: contains datablocks I. DOI: 10.1107/S1600536809036095/fk2004Isup2.hkl

Additional supplementary materials:  crystallographic information; 3D view; checkCIF report

## Figures and Tables

**Table 1:** Hydrogen-bond geometry (Å, °)

*D*—H⋯*A*	*D*—H	H⋯*A*	*D*⋯*A*	*D*—H⋯*A*
N1—H1*A*⋯O4	0.92 (4)	2.01 (4)	2.717 (4)	133 (3)
N1—H1*B*⋯O2^i^	0.95 (4)	2.14 (3)	3.016 (4)	153 (3)

Symmetry code: (i) 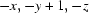.

**Fig. 1.**
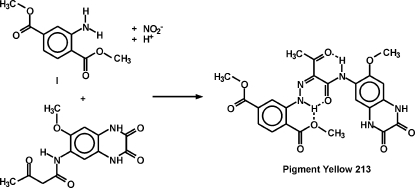
Preparation of Pigment Yellow 213 in which compound (I) is used as preproduct.

**Fig. 2.**
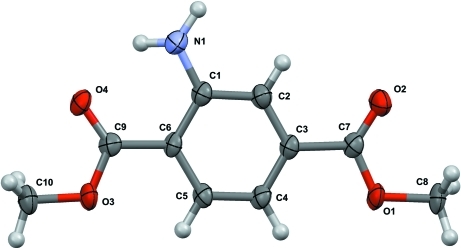
Molecular structure of compound I, with atom labels and anisotropic displacement ellipsoids (drawn at 50% probability level) for non-H atoms.

**Fig. 3.**
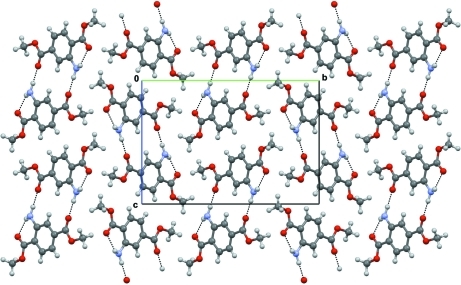
Molecular packing of compound I showing the hydrogen bond architecture; view direction [100]. The hydrogen bonds are drawn as dashed lines.

**Fig. 4.**
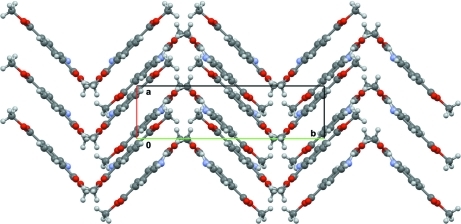
Molecular packing of compound I, showing the herringbone arrangement of the molecules. View direction [001].

## supplementary crystallographic information

### Comment

2-Aminoterephthalic acid dimethyl ester (I, Fig. 1), C_10_H_11_NO_4_, is used in the synthesis of industrial azo pigments like Pigment Yellow 213 (Metz & Weber, 1999; Stengel-Rutkowski & Metz, 2000; Herbst & Hunger, 2004; Schmidt *et al.*, 2009) (For synthesis of Pigment Yellow 213 see Fig. 1). Compound I is known since 1912 (Wegscheider *et al.*; 1912) and has been the subject of numerous investigations, *e.g*. as intermediate (Cordier & Coulet, 1994), antitumor drug (Clark *et al.*; 1995), drug against reflux disease (O'Connor *et al.*, 1999), in synthesis of pigments (Jung *et al.*, 2001; Herbst & Hunger, 2004; Schweikart *et al.*, 2007), in preparation of polymers (Lavalette *et al.*, 2002), and in parallel syntheses (Jones *et al.*, 2008); but its crystal structure has not been determined hitherto.

In order to determine the crystal structure of compound I and to search for different crystallographic phases, hydrates or solvates, a polymorph screening was performed. Different crystallization methods were used including (1) recrystallization from various solvents and solvent mixtures by heating and subsequent slow cooling, (2) overlaying a solution of the compound by an anti-solvent, (3) diffusion of an anti-solvent into a solution of the compound *via *the gas phase. The solvents included the most common organic solvents, *e.g.* dimethylsulfoxide, *N*,*N*-dimethylacetamide, *N*-methylpyrrolidone, ethers, esters, alcohols as well as acids, bases and water. According to X-ray powder diffraction no additional phases were formed.

Single crystals were obtained from technical synthesis. The structure of the molecule is shown in Fig. 2. The central six-membered ring is planar [mean deviation from best plane: 0.002 (2) Å]. The angle between the plane of the six-membered ring and the planes of the carboxy groups attached to C3 and C6 is 3.3 (2) and 5.4 (2)°, respectively. The molecules form centrosymmetric dimers connected by two symmetry-related N—H···O═C hydrogen bonds (Table 1). The second H atom of the NH_2_ group is involved in an intramolecular N—H···O═C hydrogen bond. The crystal packing is shown in Figures 3 and 4. The molecules stack along the crystallographic *a*-direction. The interplanar distance in the stack is 3.396 (2) Å. Neighbouring stacks show a herringbone arrangement.

### Experimental

The crystals of compound I were obtained from Clariant GmbH, Frankfurt am Main.

### Refinement

The H atoms attached to C atoms were geometrically positioned and were constrained using C_planar_—H = 0.95 Å, C_methyl_—H = 0.98 Å, *U*_iso_(H) = 1.2*U*_eq_(C_planar_) and *U*_iso_(H) = 1.5*U*_eq_(C_methyl_). The H atoms at the NH_2_ group were taken from a difference Fourier synthesis and were refined. The torsion angles about the O—C_methyl_ groups were refined.

### Crystal data

**Table:**

C_10_H_11_NO_4_	*F*(000) = 440
*M**_r_* = 209.20	*D*_x_ = 1.456 Mg m^−^^3^
Monoclinic, *P*2_1_/*c*	Mo *K*α radiation, λ = 0.71073 Å
Hall symbol: -P 2ybc	Cell parameters from 57 reflections
*a* = 4.7721 (12) Å	θ = 3–23°
*b* = 16.928 (5) Å	µ = 0.11 mm^−^^1^
*c* = 11.841 (5) Å	*T* = 166 K
β = 93.88 (5)°	Plate, colourless
*V* = 954.4 (6) Å^3^	0.75 × 0.32 × 0.04 mm
*Z* = 4	

### Data collection

**Table:**

Siemens SMART 1K CCD diffractometer	1687 independent reflections
Radiation source: normal-focus sealed tube	884 reflections with *I* > 2σ(*I*)
graphite	*R*_int_ = 0.140
ω scans	θ_max_ = 25.0°, θ_min_ = 2.1°
Absorption correction: multi-scan (*SADABS*; Bruker, 2000)	*h* = −5→5
*T*_min_ = 0.760, *T*_max_ = 0.995	*k* = −20→20
11829 measured reflections	*l* = −14→14

### Refinement

**Table:**

Refinement on *F*^2^	Primary atom site location: structure-invariant direct methods
Least-squares matrix: full	Secondary atom site location: difference Fourier map
*R*[*F*^2^ > 2σ(*F*^2^)] = 0.054	Hydrogen site location: inferred from neighbouring sites
*wR*(*F*^2^) = 0.134	H atoms treated by a mixture of independent and constrained refinement
*S* = 0.96	*w* = 1/[σ^2^(*F*_o_^2^) + (0.06*P*)^2^] where *P* = (*F*_o_^2^ + 2*F*_c_^2^)/3
1687 reflections	(Δ/σ)_max_ = 0.005
146 parameters	Δρ_max_ = 0.27 e Å^−^^3^
0 restraints	Δρ_min_ = −0.24 e Å^−^^3^

### Special details

**Table:**

Geometry. All e.s.d.'s (except the e.s.d. in the dihedral angle between two l.s. planes) are estimated using the full covariance matrix. The cell e.s.d.'s are taken into account individually in the estimation of e.s.d.'s in distances, angles and torsion angles; correlations between e.s.d.'s in cell parameters are only used when they are defined by crystal symmetry. An approximate (isotropic) treatment of cell e.s.d.'s is used for estimating e.s.d.'s involving l.s. planes.
Refinement. Refinement of *F*^2^ against ALL reflections. The weighted *R*-factor *wR* and goodness of fit *S* are based on *F*^2^, conventional *R*-factors *R* are based on *F*, with *F* set to zero for negative *F*^2^. The threshold expression of *F*^2^ > σ(*F*^2^) is used only for calculating *R*-factors(gt) *etc*. and is not relevant to the choice of reflections for refinement. *R*-factors based on *F*^2^ are statistically about twice as large as those based on *F*, and *R*- factors based on ALL data will be even larger.

### Fractional atomic coordinates and isotropic or equivalent isotropic displacement parameters (Å^2^)

**Table:**

	*x*	*y*	*z*	*U*_iso_*/*U*_eq_	
O1	−0.2268 (4)	0.60319 (11)	0.30114 (17)	0.0341 (6)	
O2	−0.1912 (5)	0.58551 (12)	0.11612 (18)	0.0421 (7)	
O3	0.7681 (5)	0.32502 (11)	0.44342 (17)	0.0328 (6)	
O4	0.8629 (4)	0.29952 (12)	0.26489 (18)	0.0333 (6)	
N1	0.5292 (7)	0.36927 (18)	0.0986 (3)	0.0402 (8)	
C1	0.4228 (7)	0.40860 (18)	0.1867 (3)	0.0280 (8)	
C2	0.2108 (7)	0.46595 (17)	0.1622 (3)	0.0298 (8)	
H2A	0.1487	0.4762	0.0857	0.036*	
C3	0.0944 (6)	0.50678 (17)	0.2471 (3)	0.0258 (8)	
C4	0.1783 (6)	0.49355 (17)	0.3592 (3)	0.0287 (8)	
H4A	0.0967	0.5224	0.4175	0.034*	
C5	0.3823 (6)	0.43779 (17)	0.3851 (2)	0.0277 (8)	
H5A	0.4401	0.4281	0.4622	0.033*	
C6	0.5072 (6)	0.39497 (17)	0.3004 (3)	0.0263 (8)	
C7	−0.1217 (7)	0.56897 (17)	0.2121 (3)	0.0281 (8)	
C8	−0.4282 (6)	0.66622 (18)	0.2753 (3)	0.0386 (9)	
H8A	−0.3290	0.7134	0.2514	0.058*	
H8B	−0.5277	0.6785	0.3429	0.058*	
H8C	−0.5636	0.6492	0.2142	0.058*	
C9	0.7296 (7)	0.33575 (17)	0.3320 (3)	0.0267 (8)	
C10	0.9869 (7)	0.26942 (18)	0.4801 (3)	0.0381 (9)	
H10A	0.9394	0.2170	0.4495	0.057*	
H10B	1.0030	0.2670	0.5630	0.057*	
H10C	1.1661	0.2867	0.4527	0.057*	
H1A	0.702 (9)	0.348 (2)	0.120 (3)	0.074 (14)*	
H1B	0.482 (7)	0.385 (2)	0.023 (3)	0.059 (12)*	

### Atomic displacement parameters (Å^2^)

**Table:**

	*U*^11^	*U*^22^	*U*^33^	*U*^12^	*U*^13^	*U*^23^
O1	0.0400 (15)	0.0187 (12)	0.0440 (14)	0.0105 (11)	0.0066 (11)	0.0034 (10)
O2	0.0606 (18)	0.0258 (14)	0.0392 (15)	0.0091 (12)	−0.0009 (12)	0.0034 (11)
O3	0.0391 (13)	0.0174 (11)	0.0416 (14)	0.0065 (11)	0.0000 (11)	0.0041 (10)
O4	0.0396 (14)	0.0177 (12)	0.0431 (14)	0.0031 (11)	0.0068 (11)	−0.0042 (10)
N1	0.049 (2)	0.0357 (19)	0.0361 (19)	0.0125 (16)	0.0027 (17)	−0.0045 (15)
C1	0.032 (2)	0.0169 (17)	0.0351 (19)	−0.0029 (16)	0.0028 (16)	−0.0014 (14)
C2	0.034 (2)	0.0208 (18)	0.0347 (19)	−0.0019 (16)	0.0017 (16)	0.0027 (15)
C3	0.0273 (19)	0.0148 (17)	0.0354 (19)	−0.0035 (15)	0.0038 (15)	0.0009 (14)
C4	0.037 (2)	0.0129 (17)	0.036 (2)	−0.0012 (16)	0.0058 (16)	−0.0015 (14)
C5	0.035 (2)	0.0139 (17)	0.0334 (18)	−0.0036 (15)	−0.0004 (15)	0.0010 (14)
C6	0.0305 (19)	0.0109 (16)	0.0376 (19)	−0.0015 (14)	0.0029 (16)	−0.0005 (14)
C7	0.034 (2)	0.0133 (18)	0.037 (2)	−0.0050 (15)	0.0053 (17)	0.0005 (16)
C8	0.040 (2)	0.0197 (19)	0.056 (2)	0.0112 (16)	−0.0004 (18)	0.0031 (16)
C9	0.035 (2)	0.0127 (17)	0.0329 (19)	−0.0090 (15)	0.0039 (16)	−0.0007 (14)
C10	0.041 (2)	0.0234 (18)	0.049 (2)	0.0059 (17)	−0.0031 (17)	0.0051 (17)

### Geometric parameters (Å, °)

**Table:**

O1—C7	1.330 (3)	C3—C4	1.379 (4)
O1—C8	1.455 (3)	C3—C7	1.512 (4)
O2—C7	1.195 (3)	C4—C5	1.376 (4)
O3—C9	1.333 (3)	C4—H4A	0.9500
O3—C10	1.450 (3)	C5—C6	1.403 (4)
O4—C9	1.216 (3)	C5—H5A	0.9500
N1—C1	1.365 (4)	C6—C9	1.489 (4)
N1—H1A	0.92 (4)	C8—H8A	0.9800
N1—H1B	0.94 (3)	C8—H8B	0.9800
C1—C6	1.398 (4)	C8—H8C	0.9800
C1—C2	1.418 (4)	C10—H10A	0.9800
C2—C3	1.368 (4)	C10—H10B	0.9800
C2—H2A	0.9500	C10—H10C	0.9800
			
C7—O1—C8	115.6 (2)	C1—C6—C9	120.5 (3)
C9—O3—C10	115.7 (2)	C5—C6—C9	119.9 (3)
C1—N1—H1A	111 (2)	O2—C7—O1	123.8 (3)
C1—N1—H1B	120 (2)	O2—C7—C3	124.3 (3)
H1A—N1—H1B	122 (3)	O1—C7—C3	111.9 (3)
N1—C1—C6	123.9 (3)	O1—C8—H8A	109.5
N1—C1—C2	118.4 (3)	O1—C8—H8B	109.5
C6—C1—C2	117.7 (3)	H8A—C8—H8B	109.5
C3—C2—C1	121.0 (3)	O1—C8—H8C	109.5
C3—C2—H2A	119.5	H8A—C8—H8C	109.5
C1—C2—H2A	119.5	H8B—C8—H8C	109.5
C2—C3—C4	121.3 (3)	O4—C9—O3	122.3 (3)
C2—C3—C7	117.0 (3)	O4—C9—C6	124.8 (3)
C4—C3—C7	121.7 (3)	O3—C9—C6	112.9 (3)
C5—C4—C3	118.7 (3)	O3—C10—H10A	109.5
C5—C4—H4A	120.6	O3—C10—H10B	109.5
C3—C4—H4A	120.6	H10A—C10—H10B	109.5
C4—C5—C6	121.6 (3)	O3—C10—H10C	109.5
C4—C5—H5A	119.2	H10A—C10—H10C	109.5
C6—C5—H5A	119.2	H10B—C10—H10C	109.5
C1—C6—C5	119.6 (3)		
			
N1—C1—C2—C3	179.4 (3)	C8—O1—C7—O2	−2.7 (4)
C6—C1—C2—C3	0.4 (4)	C8—O1—C7—C3	177.5 (2)
C1—C2—C3—C4	−0.1 (4)	C2—C3—C7—O2	−1.7 (4)
C1—C2—C3—C7	177.6 (3)	C4—C3—C7—O2	176.1 (3)
C2—C3—C4—C5	−0.3 (4)	C2—C3—C7—O1	178.1 (2)
C7—C3—C4—C5	−178.0 (3)	C4—C3—C7—O1	−4.1 (4)
C3—C4—C5—C6	0.5 (4)	C10—O3—C9—O4	2.2 (4)
N1—C1—C6—C5	−179.2 (3)	C10—O3—C9—C6	−178.6 (2)
C2—C1—C6—C5	−0.1 (4)	C1—C6—C9—O4	4.7 (4)
N1—C1—C6—C9	1.2 (5)	C5—C6—C9—O4	−175.0 (3)
C2—C1—C6—C9	−179.8 (3)	C1—C6—C9—O3	−174.5 (3)
C4—C5—C6—C1	−0.3 (4)	C5—C6—C9—O3	5.8 (4)
C4—C5—C6—C9	179.3 (3)		

### Hydrogen-bond geometry (Å, °)

**Table:**

*D*—H···*A*	*D*—H	H···*A*	*D*···*A*	*D*—H···*A*
N1—H1A···O4	0.92 (4)	2.01 (4)	2.717 (4)	133 (3)
N1—H1B···O2^i^	0.95 (4)	2.14 (3)	3.016 (4)	153 (3)

Symmetry codes:  (i) −*x*, −*y*+1, −*z*.
